# Introducing a Dedicated Plastic Surgery Burns Dressings Clinic in Rural Australia: Challenges and Solutions

**DOI:** 10.1111/ans.70709

**Published:** 2026-04-28

**Authors:** Jack Gerrard, Robert Toma

**Affiliations:** ^1^ Department of Plastic & Reconstructive Surgery South West Healthcare Warrnambool Victoria Australia

**Keywords:** Australia, burns, delivery of health care, first aid, humans, outpatient clinics, plastic surgery, rural health services, surgeons, wounds and injuries

Burn injuries represent a significant clinical and health‐system burden in Australia, requiring timely specialist assessment, meticulous wound care and coordinated multidisciplinary follow‐up [1, 2, 3, 4]. While major burns are appropriately managed in designated metropolitan burn centres, the majority of burns involve smaller total body surface area (TBSA) injuries that nevertheless require expert input to optimise healing and functional outcomes [1, 2, 3, 4, 5]. For rural and regional populations, geographic distance from tertiary services and workforce constraints can fragment care pathways and create inequities in access to specialist burns management [6, 7].

Rural health services in Australia face well‐documented challenges including limited specialist availability, workforce shortages and reduced access to primary and secondary care services [1, 4, 6, 7]. Rural and remote Australians experience higher rates of hospitalisation, injury, and mortality compared with metropolitan counterparts, while simultaneously encountering poorer access to health services and a relative shortage of health professionals [8, 9, 10]. Geographic isolation and workforce maldistribution further exacerbate rural health disparities and the capacity of rural services to deliver standardised care.

Warrnambool Base Hospital, part of South West Healthcare, is a 212‐bed regional hospital serving a large catchment as the closest plastic and reconstructive surgery service for south‐western Victoria, the Grampians district of Victoria and the Limestone Coast region of South Australia with a cumulative total population of 420000 [11, 12, 13, 14]. The Modified Monash Model (MM) classifies the immediate Warrnambool area as MM 3 [15]. Beyond the Warrnambool area, the remaining majority of the catchment is considered MM 4–6, defined as people living in medium rural towns of less than 15000 to very remote areas with very limited access to services [15]. Patients in this region are often located at least 3 h from the nearest tertiary burn centre in Melbourne and five and a half hours from the Royal Adelaide Hospital Burns Unit in Adelaide, highlighting substantial travel burdens when specialist care is required. Prior to the initiative described, a small number of patients were referred to a local plastic surgery post‐operative clinic that was not resourced or designed to manage such burn injuries. The majority of burns presenting locally were managed through regional/remote emergency departments, urgent care clinics, referred to tertiary burn units necessitating many hours of travel, or followed up by general practitioners and community nursing services—leading to variable and often fragmented care [1, 2, 3, 4, 5, 6].

In response, a dedicated plastic surgery burns dressings clinic was established at Warrnambool Base Hospital offering a structured outpatient model for burns follow‐up with oversight and input from a specialist plastic & reconstructive surgeon. Eligibility criteria included burns ≤ 20% TBSA, with larger burns assessed individually. Inhalation injuries, complex paediatric burns, and cases requiring tertiary interventions were referred appropriately. The clinic operates weekly for 4 h in an outpatient setting and is staffed by a plastic surgery registrar, surgical resident and a dedicated burns dressing nurse. The clinic was combined with a complex wound review clinic as this patient group also often required extended review times and enhanced nursing support.

Formal referral pathways were embedded to support broader aspects of recovery, including access to compression garment fitting with a local lymphoedema nurse, occupational therapy (including hand therapy) for functional rehabilitation, and referral for telehealth consultation with the Victorian Adult Burns Service where indicated. Nursing staff were offered dedicated burns training and upskilling in dressings and compression garment use from the closest tertiary burns centre. Over a 14‐month period, the clinic conducted 137 dressing appointments involving 47 new patients aged 20 months to 89 years. Burn mechanisms spanned scalds, contact, flame, chemical and electrical injuries, with the majority being superficial dermal burns managed conservatively. A minority required surgical debridement and split‐thickness skin grafting performed locally, facilitated by on‐site plastic surgeons and appropriate surgical equipment. Select cases utilised biodegradable temporising matrix (BTM), reflecting reconstructive techniques aligned with tertiary practice [1, 2].

Implementation presented multiple challenges. Delivering adequate analgesia in a clinic setting initially relied on patient‐held prescriptions; this was mitigated through pre‐clinic scripts and instructions facilitating pre‐dressing analgesia. Paediatric burns and procedural sedation required collaboration with paediatric services to utilise paediatric nurses and a purpose built procedural room on the paediatrics ward. Securing dedicated clinic space within a busy regional hospital necessitated negotiation and resource justification with the outpatient clinic coordinator. Sourcing and justifying the cost of optimal dressings, including silver‐based products, posed additional challenges requiring justification with evidence based practise.

Establishing referral pathways and educating local emergency departments about the clinic's scope were essential to integration, with ongoing efforts to engage general practitioners. Patient travel distances—some up to 4 h—required consideration in scheduling and highlighted broader structural access issues inherent in rural health care. Workforce constraints typical of rural Australia also influenced service delivery, aligning with broader literature on rural workforce shortages and health service limitations [6, 9, 10].

Informal patient feedback has been overwhelmingly positive. Many patients expressed gratitude for the ability to access specialist burns care locally—often within a two‐hour journey—and remarked on reduced travel costs, fuel expenses and better alignment with work and family commitments; though this feedback has not been formally quantified.

This experience indicates that a dedicated plastic surgery‐led burns dressing clinic is feasible in a rural Australian hospital and can deliver structured specialist care closer to home. While not a replacement for tertiary burn centres, such clinics may reduce unnecessary transfers, improve continuity of care, and enhance patient experience. This clinic also provides a foundation for increased collaboration between tertiary and local services by utilising virtual healthcare to collectively care for burns patients from regional or remote areas. The challenges encountered were predominantly logistical and systemic rather than purely clinical and were addressed through pragmatic, locally tailored solutions. This model could be considered by other regional centres aiming to strengthen rural burns care within existing resource constraints.

## Data Availability

The data that support the findings of this study are available from the corresponding author upon reasonable request.
